# Impacts of extreme heat on mental health: Systematic review and qualitative investigation of the underpinning mechanisms

**DOI:** 10.1016/j.joclim.2025.100446

**Published:** 2025-04-11

**Authors:** Lea Baecker, Udita Iyengar, Maria Chiara Del Piccolo, Andrea Mechelli

**Affiliations:** Department of Psychosis Studies, Institute of Psychiatry, Psychology & Neuroscience, King's College London, De Crespigny Park, London SE5 8AF, UK

**Keywords:** Extreme heat, Mental health, Lived experience, Public health, Community mental health, Environment

## Abstract

**Introduction:**

We are living on an increasingly hot planet, with negative impacts for the mental health of affected individuals and communities. A better understanding of the physiological, psychological, behavioral, and social mechanisms which underlie these impacts could inform the development of effective interventions. Here, we conducted a mixed-method study combining a systematic review and qualitative investigation to explore these mechanisms.

**Methods:**

Adhering to the PRISMA guidelines, we searched PubMed for publications related to extreme heat, mental health, and mechanisms. Peer-reviewed studies reporting original data on mechanisms explaining the impact of extreme heat on mental health were included. In addition, we conducted six focus groups on extreme heat and mental health with a total of 33 participants (*n* = 21 people with lived experience, *n* = 12 healthcare professionals). Transcripts were analyzed using thematic content analysis.

**Results:**

Out of 241 articles identified by the literature search, four studies were eligible for inclusion. These provided limited evidence for activation of the hypothalamic-pituitary-adrenal axis, increased psychological stress, reduced exercise, and sleep disruption. The analysis of the focus groups expanded these findings by highlighting additional themes related to physical (e.g., fatigue), psychological (e.g., health anxiety), behavioral (e.g., reduced access to usual coping strategies), and social mechanisms (e.g., social isolation).

**Conclusion:**

There is a dearth of reliable data on the mechanisms underpinning the impacts of extreme heat on mental health. Our mixed-method approach identified a range of promising physiological, psychological, behavioral, and social mechanisms, and highlights the importance of including people with lived experience in the conversation.

## 1 Introduction

We are living on an increasingly hot planet. Between July 2023 and June 2024, global temperatures were 1·64 °C higher than in pre-industrial times, with more frequent and more intense heatwaves being recorded in several locations [1]. While negative impacts of extreme heat on physical health have long been recognized, a more recent finding is that extreme temperatures can also have profound negative effects on mental health. An investigation based on medical records of 2·2 million citizens in the USA revealed an 8 % increase in the rate of emergency hospital visits due to mental health issues when temperatures were in the top 5 % [2]. Another investigation reported a 9.7 % increase in hospital attendance or admission for mental illness in the context of heatwaves in Australia and Vietnam, defined as daily maximum temperatures of at least 35 °C for at least 3 days [3]. These findings are backed up by an increasing number of reviews and meta-analyses reporting that high temperatures are associated with higher levels of distress, anxiety, depression, and suicide [4,5]. Emerging evidence suggests that these impacts can be fatal, with a recent meta-analysis reporting a 2·2 % increase in mental health-related mortality per 1 °C rise in ambient temperature [6].

Mental health disorders affect over 10 % of the global population [7], are a major contributor to the global disability burden [8], and reduce life expectancy by up to 20 years [9]. While the negative impacts of extreme heat on mental health are increasingly recognized, less attention has been paid to the mechanisms underpinning these impacts. Here, we use the term ‘mechanisms’ to refer to physiological, psychological, behavioral, or social changes which (i) are directly or indirectly caused by high ambient temperature, and (ii) have a negative impact on the mental health of affected individuals and communities. As there is some overlap in classification, we define the types of mechanism as follows. Physiological mechanisms involve heat-related changes in the body that influence mental health. Psychological mechanisms relate to the perception of heat, primarily through feelings of stress and anxiety, and can occur even without physical symptoms. Behavioral mechanisms involve changes in behavior due to heat, while social mechanisms refer to heat-related changes in social interactions. A better understanding of these mechanisms is important for at least two reasons. Firstly, it could inform the development of effective interventions for supporting the mental health of affected individuals and communities, and secondly, it could help clinical services allocate resources and optimize healthcare.

Here, we adopted a mixed-methods approach combining a systematic review and qualitative approach to investigate the possible mechanisms explaining the association between extreme heat and severe mental health issues. As the first systematic review on this topic, our aim was to assess the current evidence base for physiological, psychological, behavioral, and social mechanisms, and consider how this might inform the development of effective interventions for minimizing negative impacts on mental health. In light of the paucity of the current evidence base, we also conducted a qualitative investigation with two distinct groups. The first group comprised of individuals with past experiences of extreme heat, most of whom also had a history of mental illness (here referred to as ‘individuals with lived experience’). The second group comprised of mental health professionals who had past and/or current experience working with individuals with mental health issues (here referred to as ‘healthcare professionals’). The two groups participated in a series of six focus groups designed to gather lived experience insights into possible mechanisms to inform future studies.

## 2 Methods

### 2.1 Systematic review

The protocol for the systematic review followed the Preferred Reporting Items for Systematic Reviews and Meta-Analyses (PRISMA) guidelines and was registered with PROSPERO (CRD42024574080) [10]. We searched PubMed for papers published up to 15th August 2024 using search terms relating to extreme heat, mental health, and pathway or mechanism (Appendix A.1). We also conducted citation searching of systematic reviews on heat that were part of the search results. The inclusion criteria were: (1) studies reporting original data on one or more possible mechanisms or pathways explaining the impacts of extreme heat events on mental health using human or animal models; (2) articles published in peer-reviewed journals; (3) articles published in English. The exclusion criteria were: (1) studies reporting an association between extreme heat and mental health without assessing the underpinning mechanisms or pathways; (2) review articles discussing possible mechanisms or pathways without presenting original data. As this is an emerging area of research, we did not exclude studies based on potential bias (e.g., small sample size). Two authors independently conducted the search and screened articles based on the inclusion and exclusion criteria. In case of disagreement, the opinion of a third author was sought. Extracted data included article details (authors, publication year), participant information (sample size, gender, mean age, clinical status, inclusion/exclusion criteria), mechanisms under investigation (type, name), and outcome measures (clinical and functional measures). The same reviewers independently assessed risk of bias of the included studies using the Cochrane Risk of Bias Tool RoB 2 [11] for any included randomized controlled trials and ROBINS-E for non-randomized studies of exposure [12]. The results of the systematic review are presented in narrative synthesis.

### 2.2 Qualitative investigation

Participants for six focus groups were recruited from the general population (min. age 18) via the King's College London university recruitment newsletter and word of mouth. In particular, people with lived experience of mental illness and mental health professionals were encouraged to participate. Experience of mental illness was based on self-reports. Diagnostic information was not collected, as our aim was to adopt a broader, transdiagnostic approach, providing a foundation for future diagnosis-specific research. The topic guide included questions on participants’ own mental health during extreme heat, or that of service users under their care, and their thoughts on the mechanisms of the impact of extreme heat on mental health (Appendix A.2). Each focus group was facilitated by two members of the research team, lasted 1.5 h, and was audio-recorded for transcription purposes.

Verbatim transcripts were analyzed using thematic content analysis [13]. All focus groups were double-coded by members of the research team using NVivo software. A coding frame was developed after the first focus group through team consensus and then iteratively revised over the following groups. While coding was done separately for participants with lived experience and healthcare professionals, themes were developed based on the coding frames from both groups. Analysis was primarily deductive with the aim to identify physiological, psychological, behavioral, or social mechanisms, but the analysis also included inductive elements for the identification of sub-themes.

The qualitative investigation received full ethical approval by the Psychiatry, Nursing and Midwifery Research Ethics Subcommittee at King's College London (reference number LRS/DP-23/24-41409). Participants provided written informed consent.

## 3 Results

### 3.1 Systematic review

**Fig. 1:**
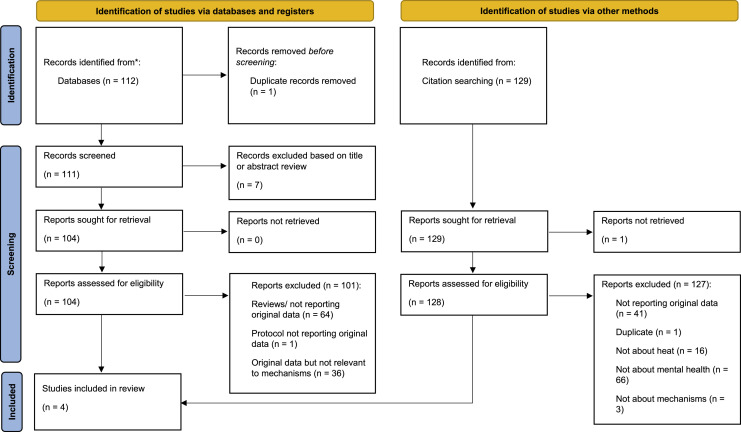
PRISMA flow diagram.

Fig. 1 shows the PRISMA flow diagram of the search results. Our search identified 112 articles, however only three of these presented original data on possible mechanisms on the impact of extreme heat on mental health. In addition, we identified one systematic review on heat [14] among the search results and conducted citation searching for its 129 references; this led to the identification of one additional article [15], resulting in a total inclusion of four articles (Table 1).

**Table 1:** Overview of the articles included in the systematic review.

Study	N	Country	Mechanism type	Mechanism name	Outcome measure(s)
Fang et al. 2023 [16]	20	China	Physiological	Activation of hypothalamic-pituitary-adrenal axis, oxidative stress, inflammation, neurotransmitters	Anxiety (measured on the State Anxiety Scale part of the Chinese version of the State Trait Anxiety Scale)
Hou et al. 2023 [15]	41,414	China	Physiological	Physical health	Self-reported mental health over the past week (based on the CESD 8)
			Behavioral	Sleep quality	Self-reported mental health (CESD 8)
			Behavioral	Sleep hours	Self-reported mental health (CESD 8)
Mullins & White 2019 [18]	>1 million	USA	Behavioral	Sleep disruption	Mental health-related emergency department visits (data from California's Office of Statewide Health Planning and Development), suicide (data from the National Vital Statistics System), self-reported mental health (survey question: “Now thinking about your mental health, which includes stress, depression, and problems with emotions, for how many days during the past 30 days was your mental health not good?”), self-reported sleep quality (survey question: “During the past 30 days, for about how many days have you felt you did not get enough rest or sleep?”), sleep quantity based on time use surveys
Zhang et al. 2023 [17]	23,393	China	Psychological	Increased stress	Self-reported mental health (survey questions: “I have as much pep as I had in 2014″; “I am as happy now as I was when I was younger”; “As I get older, things get better than I thought they would be”)
			Behavioral	Increased frequency of alcohol consumption	Self-reported mental health (see above)
			Behavioral	Reduced physical exercise	Self-reported mental health (see above)

Abbreviations: CESD 8, short version of the Center for Epidemiologic Studies Depression scale.

#### 3.1.1 Physiological mechanisms

A study by Fang et al. [16] reported some evidence for inflammatory response and hormonal changes as physiological mechanisms linking ambient temperature and mental health. The authors carried out a randomized controlled trial in 20 college students in Beijing (China) to observe the results of the effects of a heat exposure intervention on human anxiety, collecting blood samples before and after a heat exposure experiment and using metabolomic and transcriptomic approaches to quantify serum metabolites and immunoassay technology to explore antibodies, antigens, proteins and glycoproteins [16]. Even short exposure (1.5 h) to high ambient heat (32 °C) led to a significant increase in anxiety levels. The analysis of multi omics data revealed that this increase was mediated by the activation of the hypothalamic–pituitary–adrenal axis, inflammation, oxidative stress, and subsequently unbalanced neurotransmitters. Metabolites such as BDNF, GABA, and glucocorticoids released by the adrenal glands were identified as biomarkers of heat-induced anxiety. In light of the small sample size (*n* = 20) and the fact that all participants were students (age 22·45 ± 2·67 years), replication in a larger and more representative sample will be required; nevertheless, these findings provide preliminary evidence for activation of the hypothalamic–pituitary–adrenal axis, inflammation, and oxidative stress as physiological mechanisms explaining the impacts of extreme heat on mental health.

Moreover, Hou et al. [15] tested the assumption that physical health has a direct impact on mental health during extreme heat using data from a nationally representative survey (China Family Panel Studies) from 2012 to 2016. Based on self-reports of physical health over the past two to four weeks with mental health over the previous week (as assessed using the Center for Epidemiological Studies Depression 8 Scale), they reported (1) a positive but non-significant relationship between high temperature and having been severely ill, and (2) a statistically significant impact of poor physical health on poor mental health. The authors concluded that mental health during high temperatures is affected by physical health status.

#### 3.1.2 Psychological mechanisms

One of the included studies reported some evidence for stress and anxiety as a psychological mechanism leading to mental health issues. Zhang et al. [17] integrated meteorological information with the China Health and Nutrition Survey's self-assessment data from 2006 to 2015 to assess mental health impacts of extreme heat in 23,393 individuals aged 50 and above. The authors reported a significant negative association between extreme heat events and mental health. In addition, more frequent heatwaves were associated with higher levels of psychological stress, which was assumed to be a risk factor for developing mental illness over the long term and therefore considered a mechanism.

#### 3.1.3 Behavioral mechanisms

Three of the included studies examined behavioral mechanisms. Zhang et al. [17] examined reduced physical activity and increased alcohol consumption as possible behavioral mechanisms. While heatwaves were associated with a reduction in physical activity, further statistical analyses did not support this as a mechanism for worse mental health. In addition, heatwaves led to increased frequency of alcohol consumption in the respondents, which the authors considered a potential mechanism for worse mental health; however, they did not investigate this further.

Finally, both Mullins & White [18] and Hou et al. [15] explored sleep disruption as a potential behavioral mechanism. Integrating meteorological, medical and death records from the USA over the period 1960–2016 as well as self-reported mental health from phone surveys between 1993–2012, Mullins & White [18] reported that higher temperatures are associated with higher rates of mental health-related emergency visits, suicide, and worse mental health (*n* > 6 million). In further subanalyses, the authors report two pieces of evidence to support the notion that temperature-related sleep disruption is an active mechanism for the observed changes in mental health. First, warmer temperatures were associated with worse sleep quality (increases in nights of poor sleep, *n* = 1,325,562) and duration (decreases in minutes slept; *n* = 83,746); in each case the relationship was roughly linear, with colder temperatures leading to improvements in both measures. Second, changes in both emergency visits and suicides were almost entirely driven by changes in nighttime temperature (i.e., minimum daily temperature) rather than daytime temperatures (i.e., maximum daily temperature), supporting the notion of sleep disruption as an underpinning mechanism.

Hou et al. [15], following the methods described for physiological mechanisms above, also used self-reports of sleep quality and quantity to assess the association with mental health. All measures were assessed for the week prior to the survey date and linked to temperature data, controlling for humidity, wind speed, and sunshine duration. Higher temperatures significantly reduced sleep quality and quantity, and poor sleep was associated with worse mental health and higher risk of mental illness based on the scores from the Center for Epidemiological Studies Depression 8 scale.

#### 3.1.4 Social mechanisms

None of the published studies has attempted to directly assess the evidence base for any social mechanisms.

#### 3.1.5 Risk of bias assessment

For the purposes of this systematic review, the included studies were rated as high risk of bias (Appendix A.3 and A.4). The main reasons included the following: (1) measurement focused on short-term mental discomfort rather than long-term mental health issues, (2) personal exposure to temperature was not assessed, (3) mechanisms and outcome variables relied on self-reports, and/or (4) analysis protocols were not pre-published.

### 3.2 Qualitative insights

A total of 33 participants took part across six focus groups in June and July 2024 (*n* = 21 individuals with lived experience, *n* = 12 healthcare professionals) (Table 2). Our thematic content analysis identified a number of explanations for the association between extreme heat and mental health issues, which we identified as physiological, psychological, behavioral, and social mechanisms (see Table 3 for summary and illustrative quotes and the Appendix A.6 for a visual overview on all themes and sub-themes).

**Table 2:** Demographics of focus group participants. Data on gender, age, history of mental illness, and ethnicity were self-reported by the participants when they submitted their written expression of interest in participation. Further details are provided in the Appendix (A.5).

		Participants with lived experience				Healthcare professionals			
		Group 1	Group 2	Group 3	Total	Group 1	Group 2	Group 3	Total
N		8	6	7	21	3	4	5	12
Gender (N, %)	Male	1 (12.5 %)	1 (16.7 %)	3 (42.9 %)	5 (23.8 %)	0 (0.0 %)	0 (0.0 %)	0 (0.0 %)	0 (0.0 %)
	Female	7 (87.5 %)	5 (83.3 %)	4 (57.1 %)	16 (76.2 %)	2 (66.7 %)	4 (100.0 %)	5 (100.0 %)	11 (91.7 %)
	Nonbinary	0 (0.0 %)	0 (0.0 %)	0 (0.0 %)	0 (0.0 %)	1 (33.3 %)	0 (0.0 %)	0 (0.0 %)	1 (8.3 %)
Mean age (age range)		24.6 (20–36)	27.3 (22–36)*	41.9 (22–62)	31.5 (20–62)*	44.3 (26–56)	26.0 (23–29)⁎⁎	31.6 (28–39)	33.5 (23–56)⁎⁎
% with current or past mental illness		62.5 %	83.3 %	71.4 %	68.2 %	0 %	25.0 %	20.0 %	16.7 %
% working in mental health care⁎⁎⁎		NA	NA	NA	NA	66.7 %	50.0 %	100.0 %	75.0 %
Ethnicity (N, %)	Caucasian	1 (12.5 %)	3 (50.0 %)	2 (28.6 %)	6 (28.6 %)	2 (66.7 %)	2 (50.0 %)	3 (60.0 %)	7 (58.3 %)
	Asian	5 (62.5 %)	1 (16.7 %)	4 (57.1 %)	10 (47.6 %)	0 (0.0 %)	1 (25.0 %)	1 (20.0 %)	2 (16.7 %)
	Black	0 (0.0 %)	0 (0.0 %)	0 (0.0 %)	0 (0.0 %)	0 (0.0 %)	1 (25.0 %)	0 (0.0 %)	1 (8.3 %)
	Other	1* (12.5 %)	1* (16.7 %)	1 (14.3 %)	3⁎⁎ (14.3 %)	1 (33.3 %)	0 (0.0 %)	1 (20.0 %)	2 (16.7 %)
Mode of focus group		In-person	Virtual	Virtual	NA	Virtual	Virtual	Virtual	NA

Abbreviations: NA, not applicable.

⁎ Data missing for one participant who selected ‘Prefer not to say’.

⁎⁎ Data missing for two participants who selected ‘Prefer not to say’.

⁎⁎⁎ The three healthcare professionals that were not mental health professionals had experience of supporting patients with mental health-related issues as part of their practice.

**Table 3:** Themes and sub-themes identified in the thematic content analysis of the focus group transcripts with supportive quotes from the participants.

Theme	Sub-theme	Sub-sub-theme
Physiological mechanisms	Direct link between physical and mental wellbeing. Healthcare professional: *"I think there's always some physiological aspects because when it's really hot, you're going to sweat more and you might start having a lot of migraines or headaches due to the weather also. And that might make you just really hot and bothered."*	Symptoms caused by dehydration. Healthcare professional: *“I was thinking about someone I was working with as a support worker years ago, who was living on her own and she was quite an elderly lady but she was really severely dehydrated and she had an episode of delirium I think. We had to take her to A&E. […] I think her condition had deteriorated. And it was quite severe, we didn't know what was happening.”*
		Discomfort of sweating. Person with lived experience: *"I don't like the sensation of sweating. You know, it makes me feel dirty and uncomfortable, so I'm already kind of out of my comfort zone."*
	Diversion of energy away from everyday functions. Person with lived experience: *"I think the human body uses more energy to try and handle the heat. Basically, to just keep your organs going and your systems going as they normally would, I think it just requires more energy in extreme heat, and I think that obviously has an impact on the way your brain is going to work or react to things, like the usual things. What happens on a day-to-day might feel like - they might feel catastrophic even, or you know, because you're more irritable, because you don't feel like you've got enough energy to deal with those things."*	Lack of energy and fatigue. Person with lived experience: *"I don't want to do anything today [on a hot day] - but not relaxed, but like I cannot do anything. I just want to sit down and not move. And maybe that's because when you move, your body is producing heat. So that's something bad. You don't want to move, but because you're not moving, you're depressed."*
		Reduced emotional regulation. Person with lived experience: *"I just seem a lot moodier on those days. And then I get angry with things more easily and you know, towards the end of the day, […] mental health wise, I feel like I've been a bit maybe too harsh of a person or a bit too unfair in my reactions towards others. And then I start to kind of beat myself up over it. Because I feel like maybe I could have tried harder to control myself. But it is hard when it comes to biological reactions within you. It's hard to handle that. It's hard to handle nature if that makes sense."*
		Reduced cognitive function. Person with lived experience: *"Brain fog is a huge issue. Irritability is one thing, but the irritability comes from the fact that I'm unable to function as I may be.”*
Psychological mechanisms	Increased stress and anxiety.	General feelings of anxiety related to overheating. Healthcare professional: *“When you're hot, you're just more easily stressed, like anything that might trigger you normally is a bigger trigger. And yeah, I guess it just leads people to just become more upset more easily and adds more stress. […] And I think especially if we're also thinking about clients’ ability to kind of attend clinic in person and if they're going on public transport or something - like no one's going to really want to travel on the bus or on the train in really, really hot weather.”* Person with lived experience: *"[The news] are treating it like it's going to be this […] disastrous thing. Like 'It is over, […] the sun is going to come out and you're going to die' and they're like, 'don't open your window' – it's just, it's so negative. And I think that in itself puts your expectations in this place. […] Like after that heatwave [in London in 2022], it was the most negative experience as well, just because the way in which I'd open my phone and it'd be like 'people are dying'. It just doesn't set the right mindset."*
		Health anxiety. Healthcare professional: *"Especially people with health anxiety, they then worry about the effects that the heat will have on their health. And I definitely had instances as well, where clients have been really, really stressed about how much water they've had today or the possibility of them fainting in the weather."*
		Embarrassment of sweating and body odor. Person with lived experience: *"I sweat more like my upper body than my lower body. So then that causes some issues because of like, you know, anxiety and feeling self-conscious [about sweat stains or body odor]. […] I think it would be that would affect mental health."*
	Increased mental load from caring responsibilities. Person with lived experience: *"I used to live with my parents and my grandma lived with us. And obviously, like elderly people like certain health groups are at higher physical risk, up from the heat, so it can cause death. It can cause, like, serious health problems. So, I mean, I don't think my grandma was that worried about it, but because my mum was caring for her that was like an extra worry in the back of her mind. Like, we need to keep the house cool, make sure she stays hydrated and eats properly. Otherwise something serious could happen. So if there is any extra stress, that can impact your mental health as well."*	
	Lack of control over the situation. Person with lived experience: *"I think for me at least, that's where I would guess the agitation, frustration, stress comes from is that it's that the locus of control is outside of you. So if you don't have spaces that have A/C or a fan, if you don't have a way to change it - like [participant name] said, you just remember feeling it was unbearable - you don't have the ability to change that, that brings about helplessness almost."*	
Behavioral mechanisms	Sleep disruption. Person with lived experience: *"The effect it's got on me is that I also can't sleep well. I can't fall asleep. Or if I finally fall asleep when I wake up, I feel like a vegetable. And like I can't get myself to get out of bed at the times I normally get up. I don't just feel sluggish [..]. And then overall when it comes to my to-do list, I'm just not able to start doing things. I just feel like I want to keep sitting down on different surfaces. I get up from the sofa and then I want to sit on the chair and then I get up from the chair and I want to sit back on the sofa, that sort of thing."*	
	Reduced access to usual coping strategies, incl. less exercise. Healthcare professional: *"I couldn't go and do anything because it was actually just way too hot to even really move, and which then obviously results in people kind of just spending the day at home. Curtains closed, maybe watching TV, like not really engaging with anyone face to face, probably. Which then does just increase socialization, probably increases loneliness and anxiety in relation to that as well."* Person with lived experience: *"If I cannot do sport for two or three or four days […], it impacts a lot on my mental health."*	
	Increased substance use. Healthcare professional: *"I would say most people drink alcohol and especially in hot weather, sitting out in the garden. And it does have an effect, especially if people overdo it."*	
	Less engagement with therapeutic treatment. Healthcare professional: *"Less attendance is the first thing that just comes to my mind. […] Motivation levels drop and I just find that there are more challenges to people actually attending their sessions. And if they do attend, I just do notice a difference in motivation levels and that there's more a desire to kind of focus is on just trying to regulate body temperature and find comfort rather than actually being able to look at any anything on a deeper level. It kind of reminds me a bit of Maslow's hierarchy of needs that actually my body is just not comfortable, I'm not able to think about anything on a deeper psychological level right now, because there's something else going on that's more pressing."*	
Social mechanisms	Social isolation. Healthcare professional: *“I remember the 2022 heatwave, […] I remember that I spent the whole day inside with the curtains closed. […] And I felt quite socially isolated actually because there was no one else at home. […] I couldn't go and do anything because it was actually just way too hot to even really move, and which then obviously results in people kind of just spending the day at home. Curtains closed, maybe watching TV, like not really engaging with anyone face to face probably, which then does just increase socialization, probably increases loneliness and anxiety in relation to that as well.”*	

While the majority of participants thought the negative impact of extreme heat on their mental health was due to its physical impact - people with lived experience in particular struggled to imagine any other mechanism - further discussions highlighted the interplay of physical and non-physical factors. One healthcare professional likened the question of mechanisms to a *“chicken and egg"* situation, where *“it all intertwines and whichever lens you're looking at it through first [physiological, psychological, behavioral, social], you can almost use that as the base”*.

#### 3.2.1 Physiological mechanisms

Heat-induced physical discomfort, symptoms, or even illnesses were thought to have direct negative effects on mental wellbeing. One individual with lived experience described this effect with the following analogy: *“It's the same reason if you're stubbing your toe, you're just going to get annoyed because it's like a physical discomfort that will lead to frustration or anger or annoyance […]. If I'm in a physical discomfort, that translates almost immediately into a mental discomfort.”*

An important direct link between physical and mental wellbeing mentioned by participants, especially healthcare professionals, was through dehydration. They thought that dehydration can lead to headaches or dizziness, which might in turn be associated with cognitive impairment, irritability, and anxiety. Additionally, while sweating is a less severe physical symptom, participants repeatedly noted that sweating makes them uncomfortable, irritable, and less likely to engage in social situations.

Participants also felt that the body requires more energy for physical functions during extreme heat (e.g., thermoregulation) and therefore diverts resources away from the mental functions needed to manage everyday stressors. The majority felt that lack of energy and extreme fatigue made them unable to function the way they normally would. They thought that this lack of energy went hand in hand with reduced cognitive function. Furthermore, participants speculated that brain fog was the primary cause of reduced motivation, concentration, and productivity, which many considered to be the main triggers for their increased frustration and irritability during heat.

In general, participants felt less able to regulate their emotions during extreme heat, as evidenced by irritability, overwhelm, moodiness, even anger and aggression. Participants thought this may be caused by the general overwhelm of the physical senses and/or the impact of extreme heat on hormones. As one healthcare professional explained, “*you might start feeling flustered, overwhelmed with the work because not only are you dealing with the work itself but your own senses. And your physical feelings are experiencing something that's not our usual basis for a comfortable environment”.*

#### 3.2.2 Psychological mechanisms

Participants felt that extreme heat can increase feelings of stress and anxiety, especially in people who already struggle with mental health difficulties. They reported different kinds of anxiety: (1) generally feeling more anxious, potentially exacerbated by sensationalist media coverage, (2) health anxiety about experiencing physical symptoms, and (3) anxiety and embarrassment about sweating and body odor around other people. In addition, a few participants noted the increased mental load and stress levels from caring responsibilities during extreme heat, as children and elderly are seen as more vulnerable.

People with lived experience attributed their negative mental wellbeing to a perceived lack of control over the situation, especially when they lacked access to cool spaces for temporary relief or when the heat persisted for several days. One participant recalled the 2022 heatwave in London, saying *“I just remember it so distinctly because it was like four days of just pain essentially. […] I don't think I've ever felt so helpless.”*

#### 3.2.3 Behavioral mechanisms

Almost all participants reported worse sleep quality and quantity during extreme heat and highlighted this as a primary mechanism for their poor mental health. They speculated that poor sleep would lead to fatigue, lack of energy, anxiety, irritability, and depressive moods, and it would therefore compound the physiological mechanisms discussed above. One individual with lived experience described it in the following way: "*Your room just becomes like a greenhouse. And because of that, you'd struggle to sleep. And then you have worse sleep and you wake up feeling just so much worse. Then you don't want to do anything. And it just sets the mood of not wanting to get out of bed."*

Furthermore, extreme heat often reduces people's access to their usual coping strategies, such as exercising, going for a walk, or meeting friends, because people stay indoors to keep physically safe from the heat. One healthcare professional referred to these as *“preventative strategies […] that would usually kind of keep their mental health stable”*, so the adverse effects of not being able to engage in these are apparent.

A few participants noted that the hot weather, and potentially heat-induced discomfort, may lead people to consume more alcohol or other substances. Adding to the direct physical health risks (including dehydration), one healthcare professional specifically pointed out that people may become less receptive to harm reduction measures when they are physically overwhelmed by the heat.

A notable behavioral risk highlighted by healthcare professionals is reduced engagement with therapeutic treatment. Physical discomfort may make people less motivated to adhere to their treatment schedule, such as attending therapy sessions or taking their medication, which may have detrimental short- and long-term consequences for their mental health.

#### 3.2.4 Social mechanisms

Staying indoors to keep physically safe from the heat can often lead to less social interaction and increased social isolation, which may cause feelings of loneliness and depressive moods. Almost all participants perceived this as a major risk during a heatwave. One individual with lived experience described their behavior in the following way: *"When it's hot over the summer period, I try and stay away from places. […] I just sort of retreat […]. And for me that's probably an issue that I retreat too much and then I don't socialize at all."* A few participants also drew the comparison to experiences during the COVID-19 pandemic, where people living alone were seen as particularly vulnerable to mental health issues.

## 4 Discussion

As the association between extreme heat and mental health is now well recognized in the scientific literature, an increasing number of articles are discussing possible mechanisms. We conducted the first systematic review on this topic. While the vast majority of existing articles on extreme heat and mental health discuss possible mechanisms, our systematic review highlighted a dearth of actual data on this topic. Only four studies have attempted to test possible mechanistic explanations using original data.

One study, which focused on possible physiological mechanisms, identified the activation of the hypothalamic-pituitary-adrenal axis and the presence of inflammation and oxidative stress as the primary pathways through which heat exposure contributes to anxiety [16]. There was also some evidence that poor physical health itself has a direct impact on mental health [15]. Another study, which focused on possible psychological mechanisms, provided some support to the notion that higher levels of psychological stress could be an active mechanism for the subsequent onset of mental illness [17]. The same study also reported increased alcohol consumption as a potential mechanism [17]. It was assumed, rather than demonstrated, that higher levels of psychological stress or alcohol consumption would increase the risk of developing mental illness over the long term. Two further studies, which focused on possible behavioral mechanisms, provided support to the notion that temperature-related sleep disruption is an active mechanism for the observed changes in mental health [15,18].

**Fig. 2:**
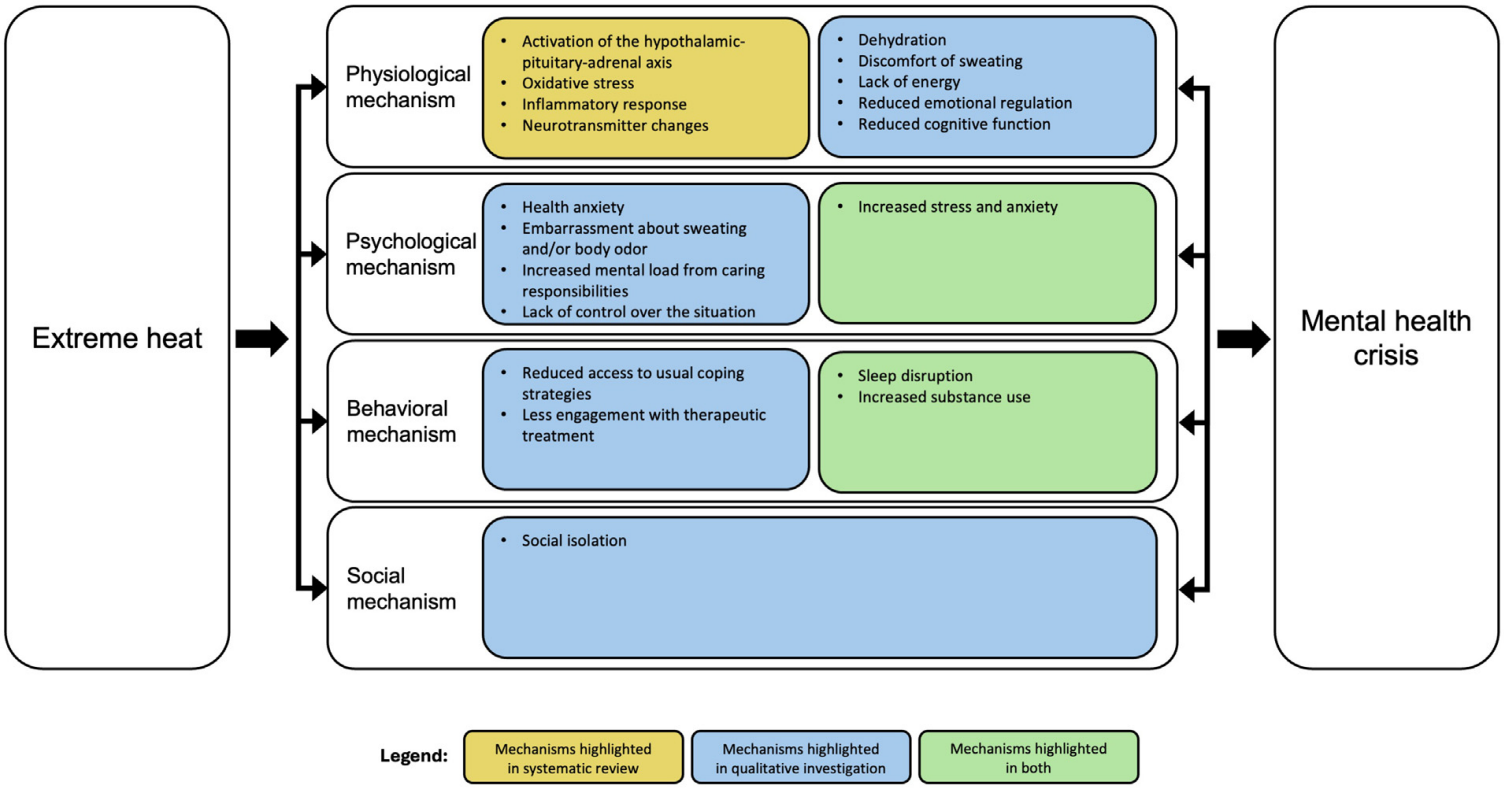
Multi-level framework for explaining the impacts of extreme heat on mental health based on our mixed-method study. The bidirectional arrows amongst different mechanistic categories indicate the interplay amongst physiological, psychological, behavioural, and social changes.

Taken collectively, our findings indicate a lack of evidence base for mechanistic explanations of the impacts of extreme heat on mental health. The four included studies provided suggestive rather than definite evidence. Specifically, these studies revealed heat-induced physiological, psychological, and behavioral changes which are thought to increase risk of mental illness (e.g., sleep disruption). However, none of these studies assessed whether these changes led to actual mental illness. In addition, none of the existing studies included data from people with a pre-existing mental health condition, even if the impacts of extreme heat are thought to be greater in this vulnerable group [5]. Furthermore, none of the existing studies have considered social mechanisms or examined two or more mechanisms (e.g., physiological and psychological). A full understanding of how extreme heat can precipitate a mental health crisis will require a multi-level mechanistic explanation, including the interplay amongst heat-induced physiological, psychological, behavioral, and social changes (Fig. 2). A final consideration is that the vast majority of the existing studies have used a retrospective design which did not allow for a rigorous examination of possible mechanisms, with one exception which used a randomized controlled design to examine physiological mechanisms [16].

In order to inform and stimulate future research in this area, we carried out six focus groups with people with lived experience and healthcare professionals. The results of our thematic content analysis provide support to the physiological (heat-induced anxiety, direct impact of physical illness), psychological (increased stress), and behavioral (sleep disruption) mechanisms identified in our systematic literature review, and highlight potential mechanisms not investigated before. Some of these are: (1) physical mechanisms, such as lack of energy, reduced emotional regulation, and reduced cognitive function, which together led to depressive moods, irritability, and frustration; (2) psychological mechanisms, such as health anxiety, potentially leading to spiraling mental health; (3) behavioral mechanisms, such as lack of access to usual coping strategies, and disengagement with therapeutic treatment, with potentially detrimental short- and long-term effects; and (4) social mechanisms, such as social isolation, associated with depressive moods. Most of these mechanisms have been speculated about in the literature without direct evidence for the mental health effects. For example, the association between heat and sleep disruption is well-established [14], and potentially also mediates the negative effect of heat on cognitive performance [19], so our qualitative investigation lends support to the idea that both sleep disruption and reduced cognitive performance can have severe mental health impacts. Furthermore, a recent qualitative study identified an increase in various types of anxiety similar to our findings, including worries about physical symptoms, confinement (staying indoors), or the uncertainty of the situation [20]. It is also worth noting that various studies and reviews discuss the mechanisms behind the mental health impacts of climate change in general. Even though they might not specifically look at heat, some of the mechanisms discussed there, such as increased substance use [21], overlap with our findings.

### 4.1 Limitations

While the systematic review and qualitative investigation addressed a clear gap in the literature, this study had a number of limitations. Firstly, the systematic review only included four studies and all of them were at high risk of bias, thus limiting the usefulness of the findings and highlighting the need for further research. Secondly, the demographic characteristics of our sample may limit the generalizability of the results. Specifically, the sample size was relatively small, predominantly female, university-educated, and mostly based in London, UK. It is important to acknowledge that other countries tend to experience extreme heat more frequently and intensely, meaning that our findings may not fully apply to other contexts. Additionally, the location of our focus groups may have resulted in the exclusion of potential mechanisms that might be relevant elsewhere, such as the loss of farming income. Nevertheless, it is worth noting that approximately half of our participants reported having grown up abroad and/or lived in hotter countries, which allowed our focus groups to capture a diverse range of lived experiences. It is also worth considering that not all participants had a history of mental illness, and all health impacts were based on self-reports; therefore, the distinction between worse mental wellbeing and severe mental health issues induced by heat was not always clear.

### 4.2 Recommendations

Our mixed-method approach has identified several promising physiological, psychological, behavioral, and social mechanisms, highlighting the importance of including people with lived experience in the conversation. Future studies must employ a prospective design to collect more rigorous data and include people with lived experience who are at greatest risk of experiencing a mental health crisis due to extreme heat. The results of these studies could inform the development of effective interventions for supporting the mental health of affected individuals and communities. For example, supporting evidence for sleep disruption as a possible behavioral mechanism would provide a rationale for adopting increased ventilation at nighttime to minimize mental health impacts. Furthermore, a mechanistic understanding of the impacts of extreme heat on mental health could inform the development of predictive models for allocating clinical resources and optimizing healthcare in the hottest months.

## Funding

This work was supported by a Wellcome Climate Impacts Award (Grant Ref: 228033/Z/23/Z).

## Role of the funding source

The funder of this study had no role in study design, data collection, data analysis, data interpretation, or writing of the report.

## Data sharing agreement

Data are available upon request.

## CRediT authorship contribution statement

**Lea Baecker:** Writing – review & editing, Writing – original draft, Visualization, Project administration, Methodology, Investigation, Formal analysis, Data curation. **Udita Iyengar:** Writing – review & editing, Methodology, Investigation. **Maria Chiara Del Piccolo:** Writing – review & editing, Methodology, Formal analysis. **Andrea Mechelli:** Writing – review & editing, Writing – original draft, Visualization, Supervision, Methodology, Funding acquisition, Conceptualization.

## Declaration of competing interest

The authors declare that they have no known competing financial interests or personal relationships that could have appeared to influence the work reported in this paper.

## Appendix Supplementary materials

Image, application 1
