# Supplementary material for: Impacts of extreme heat on mental health: Systematic review and qualitative investigation of the underpinning mechanisms
Source: J Clim Chang Health. 2025 Apr 11;22:100446. doi: 10.1016/j.joclim.2025.100446 (PMC12851232; doi:10.1016/j.joclim.2025.100446)
Supplement: Supplementary file 1 [file mmc1.docx]

**Appendix**

**A.1.** Search terms in PubMed.

("extreme heat" OR "ambient heat" OR "extreme temperature" OR heatwave OR "ambient temperature" OR "climate change") AND ("mental health" or "mental illness" or "mental wellbeing") AND (pathway OR mechanism OR explanation)

**A.2.** Semi-structured topic guide used in the focus groups. This topic guide was developed based on a preliminary literature search and the aims of the wider research project.

1. [For mental health professionals only:] How does extreme heat affect your clinical practice?
2. How does extreme heat affect you, specifically your mental health? [For mental health professionals: How does extreme heat affect the service users under your care, specifically their mental health?]
3. What do you think are the main explanations for the effects of extreme heat on mental health? Does extreme heat influence mental health through physical changes in your body, through changes in your everyday behaviour, through changes in your surrounding environment, or something else?
4. What are risk factors that make someone more likely to experience mental health issues during extreme heat?
5. What are protective factors that make someone less likely to experience mental health issues during extreme heat?

**A.3.** Risk of bias assessment for the included Randomized Controlled Trial using version 2 of the Cochrane tool for assessing risk of bias in randomized trials (RoB 2). Figure created using robvis [1].


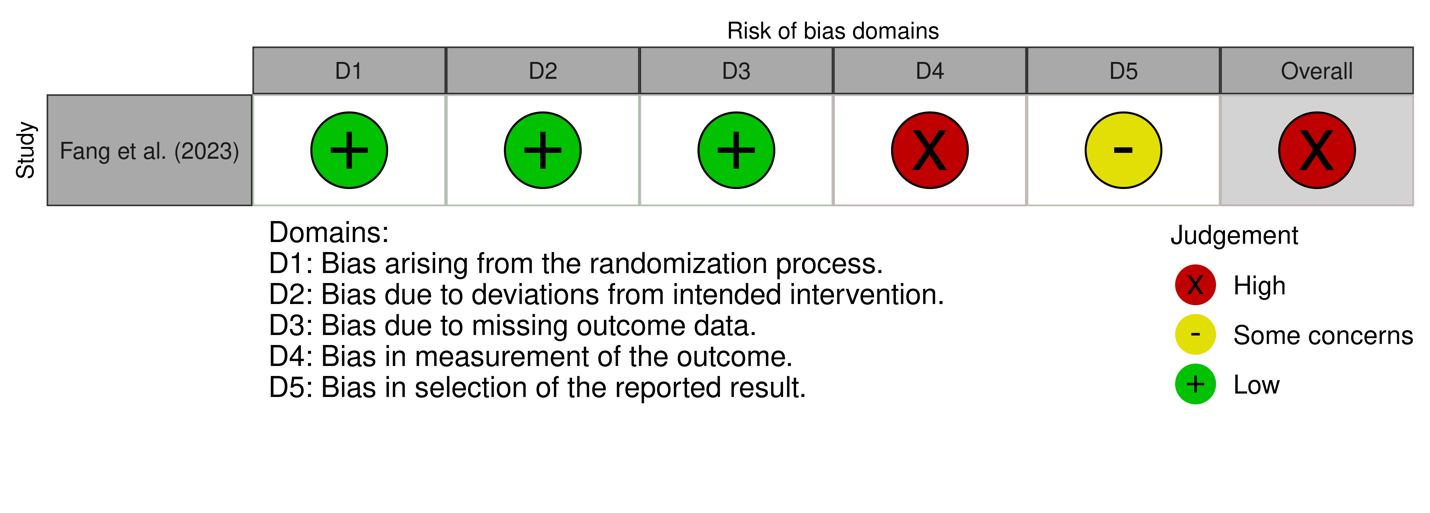


**A.4.** Risk of bias assessment for the included observational studies using Risk Of Bias in Non-randomized Studies – of Exposures (ROBINS-E). Figure created using robvis [1].


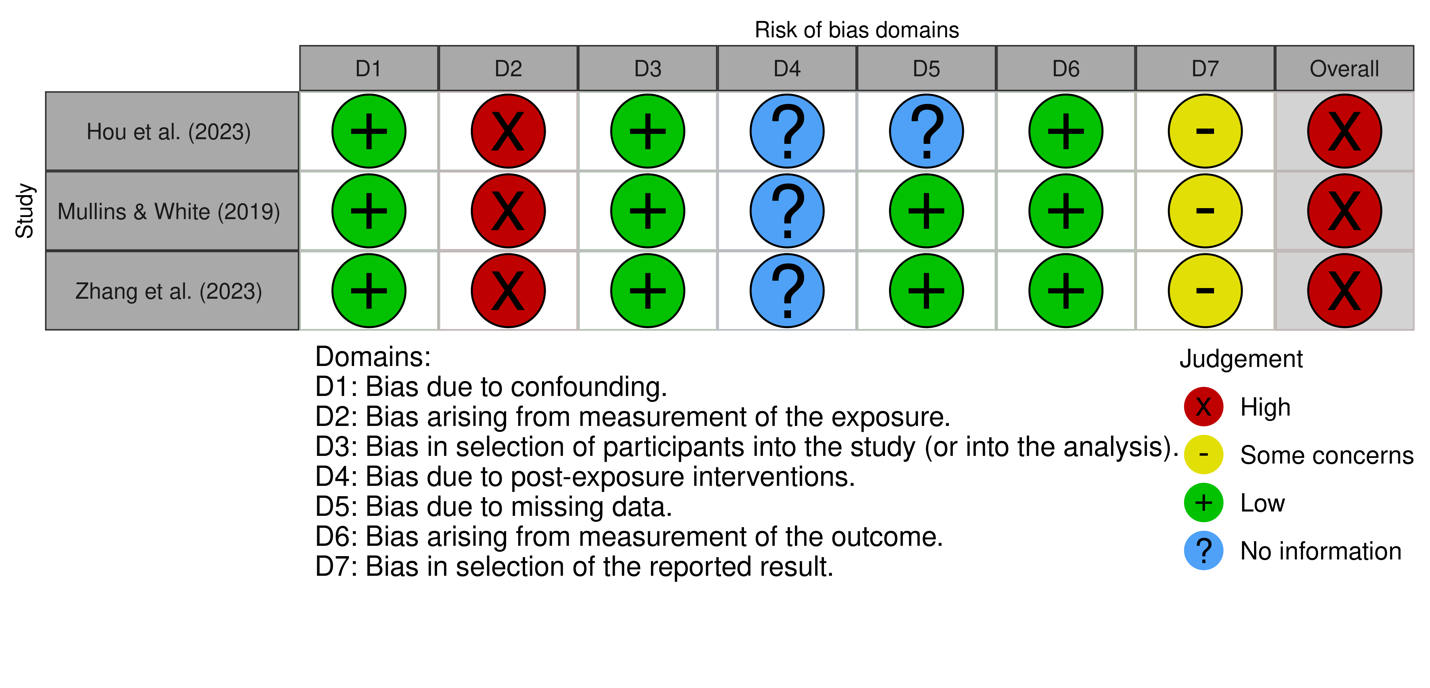


**A.5.** Further details on the healthcare professionals taking part in the focus groups.

Nine of the participants in the healthcare group directly worked in the field of mental health with the following job roles: psychotherapist, art psychotherapist, psychologist, psychiatrist, clinical psychologist, trainee clinical psychologist (n=2), substance misuse worker, support worker. The three healthcare professionals who were not mental health professionals (nurse, healthcare assistant, occupational therapist) had experience of supporting patients with mental health-related issues as part of their practice.

**A.6.** Mind map of the results of the thematic content analysis of the qualitative investigation.

**References**:

[1] McGuinness, LA, Higgins, JPT. Risk-of-bias VISualization (robvis): An R package and Shiny web app for visualizing risk-of-bias assessments. Res Syn Meth. 2020; 1- 7. <https://doi.org/10.1002/jrsm.1411>
